# Secondary Complementary Balancing Compressive Imaging with a Free-Space Balanced Amplified Photodetector

**DOI:** 10.3390/s22103801

**Published:** 2022-05-17

**Authors:** Wen-Kai Yu, Ying Yang, Jin-Rui Liu, Ning Wei, Shuo-Fei Wang

**Affiliations:** 1 Center for Quantum Technology Research, School of Physics, Beijing Institute of Technology, Beijing 100081, China; 3120201526@bit.edu.cn (Y.Y.); liujinruibit@163.com (J.-R.L.); 3120201518@bit.edu.cn (N.W.); 3120195769@bit.edu.cn (S.-F.W.); 2 Key Laboratory of Advanced Optoelectronic Quantum Architecture and Measurement of Ministry of Education, School of Physics, Beijing Institute of Technology, Beijing 100081, China

**Keywords:** single-pixel imaging, imaging system, optical imbalance, complementary balanced measurement, optical signal processing

## Abstract

Single-pixel imaging (SPI) has attracted widespread attention because it generally uses a non-pixelated photodetector and a digital micromirror device (DMD) to acquire the object image. Since the modulated patterns seen from two reflection directions of the DMD are naturally complementary, one can apply complementary balanced measurements to greatly improve the measurement signal-to-noise ratio and reconstruction quality. However, the balance between two reflection arms significantly determines the quality of differential measurements. In this work, we propose and demonstrate a simple secondary complementary balancing mechanism to minimize the impact of the imbalance on the imaging system. In our SPI setup, we used a silicon free-space balanced amplified photodetector with 5 mm active diameter which could directly output the difference between two optical input signals in two reflection arms. Both simulation and experimental results have demonstrated that the use of secondary complementary balancing can result in a better cancellation of direct current components of measurements, and can acquire an image quality slightly better than that of single-arm single-pixel complementary measurement scheme (which is free from the trouble of optical imbalance) and over 20 times better than that of double-arm dual-pixel complementary measurement scheme under optical imbalance conditions.

## 1. Introduction

Single-pixel imaging (SPI) can acquire the object information from sequential singlepixel measurements of the superposition of the modulated patterns and the object. The single-pixel detection modality offers more possibilities for the wavelengths where pixelated detectors are technically unavailable or too expensive, such as X-ray, infrared, and terahertz wavelengths. This promising indirect imaging technique [1,2,3], ever since it was proposed, has been intensively studied [4,5] and provided countless novel image measurement ideas in science and engineering fields, including quantum entanglement [6], polarimetric imaging [7], three-dimensional tracking [8], hyperspectral imaging [9], fluorescence microscopy [10], medical imaging [11], compressive holography [12], imaging through scattering media [13], optical encryption [14], etc. Most of them were motivated by compressed sensing (CS) [15,16,17] and ghost imaging [18,19] algorithms.

In SPI setups, the spatial modulation or structured illumination can be performed by using fast spatial light modulators (SLMs) such as a modern digital micromirror device (DMD) with a fast switching frequency up to 22 KHz. To our knowledge, the modulation speed can be further increased by applying structured illumination with LED arrays [20], but at low spatial resolution. According to the nature of SPI, a lot of patterns are needed for per single-frame image acquisition. Given that the response frequency of the single-pixel detector is much faster than the modulation speed of the used SLM, the latter and the number of measurements place an upper bound on the acquisition speed of the system. The modulation speed is limited by devices and technologies, and is too hard to be increased. Any little increase in modulation speed comes at a huge cost. Therefore, to achieve imaging speed beyond the regular limit, many efforts are focused on improving the sampling efficiency by optimizing the ordering of the Hadamard basis patterns [21,22,23,24] or using Fourier basis patterns [25]. Apart from these deterministic patterns, random patterns are also very popular in conventional SPI due to their simplicity and easy implementation. However, no matter which kind of the above methods is adopted, the measurements are susceptible to unfavorable ambient light, and the non-negativity of the patterns will produce a direct current (DC) component in the measured signal that cannot be ignored, leading to the degradation of reconstruction quality. Furthermore, considering that the overlap between each pattern and the object part is basically random, the system will lose half flux of photons on average.

As we all know, each micromirror on the widely used DMD can rotate about a hinge and orientates $\pm 12^{\circ }$ with respect to its normal direction, corresponding to “on” (1) and “off” (0) binary states. Thus, the light will be reflected in two directions depending on the modulation matrices loaded onto the DMD. When we look at the DMD along the opposite of these two reflection directions, only the mirrors in the “on” (“off”) state will reflect the light while the ones in the “off” (“on”) state will be dark; thus, in two reflection arms during each modulation, we will see two patterns that are exactly complementary. Given this, double-arm dual-pixel complementary differential (or positive-negative) measurement was proposed and proven to improve the measurement signal-to-noise ratio (SNR) and to make full use of all photon flux by setting two single-pixel detectors in both reflection directions [26,27]. It is also proven that it can be combined with Fourier SPI to gain good robustness to noise and to acquire better imaging performance [28]. One could also make the DMD modulate one pattern immediately followed by its inverse (complementary) pattern and take the difference between adjacent single-pixel measurements to achieve a similar effect [29,30,31,32,33]. We call this method single-arm single-pixel complementary differential measurement. By using the above two methods, the DC term of the measured values can be eliminated, while the alternating current (AC) term that responds to the fluctuations of the measurements can be retained to recover the object image of interest. Moreover, the sampling ratio can be shortened to a much lower level than the traditional SPI when obtaining the same image quality, and the immunity to environmental light and temperature drift of light source can also be enhanced. If the double reflection arms of the DMD can be used, it will save more time in the measurement compared with single-arm complementary SPI. In addition, in recent years, it has been found that introducing two or more single-pixel detectors in SPI schemes can be helpful for greatly improving imaging performance. Successful examples include the full-color imaging setup [34], simultaneous visible and infrared video system [35], which have been proven to enrich the imaging functions. As we know, a balanced detector is an integration of two separate single-pixel detectors, which can be directly used in the above-mentioned double-arm complementary SPI scheme to perform complementary differential measurement [36,37]. The balanced detector can directly subtract the two optical inputs recorded by its two photodiodes (which have better sensitivity consistency and response time consistency) and output the differential signal, realizing the cancellation of common mode noise. Actually, both dual-pixel detection and balanced detection belong to double-arm complementary measurement modality, and the use of balanced measurements in optical experiments can be traced back to the late 1960s [38]. In the above SPI schemes with balanced detection, if the balanced detector is in fiber-coupled design, the inputs need to be fiber coupled to the photodiodes of the balanced detector with the help of two identical fiber collimating elements [36]. The use of two optical fibers makes the setup flexible, but it incurs a problem of coupling efficiency, and it is difficult to ensure that the coupling efficiencies in two arms are consistent. If the balanced detector is in free-space design, multiple optical elements such as lenses and plane mirrors need to be used to make the optical signals from two reflection arms of the DMD enter into the surface of the photosensitive elements [37]. By using any of these two types of balanced detectors, once the light path changes slightly, the optical fibers need to be re-coupled or lenses need to be adjusted, which are very troublesome. Moreover, the ambient light, luminous flux loss, propagation distance, and collection efficiency in both arms are difficult to keep consistent or on the same level, thus the optical imbalance problem existing in these two arms is unavoidable.

To summarize, single-arm single-pixel complementary differential measurement can avoid the asymmetry problem that exists in the double-arm complementary SPI schemes, but the former’s sampling efficiency is much lower than that of the latter due to the failure to make full use of the reflected light from two arms. However, in the latter, regardless of using discrete detectors or balanced detectors, the optical imbalance problem existing in two reflection arms of the DMD is unavoidable, even with the use of industrialized optomechanical system design. How to eliminate the influence of optical imbalance on imaging results in double-arm complementary SPI schemes and suppress the measurement noise to the lowest level are the biggest difficulties and great challenges.

In this work, we propose a simple secondary complementary balancing mechanism on the basis of a complementary SPI scheme with balanced detection. It will sequentially encode the complementary pattern pairs onto the DMD and compute the differential signal of the outputs of the balanced detector within each complementary modulation pair. By this means, the impact of the optical imbalance in double reflection arms of the DMD will be minimized. The DC term of the measured values can be eliminated to the maximum extent, and the fluctuations of the measurements that are really useful for image reconstruction will be amplified, thus leading to the increase of the measurement SNR and accuracy. In addition, the full dynamic range of the detector can be used to record the positive-negative fluctuations. The feasibility and superiority of this method will be demonstrated through both numerical simulations and optical experiments.

## 2. Methods

In SPI, the single-pixel detector records the inner product (superposition) of the object image O(c,d) and each modulated pattern $P_{i}\left(c,d\right)$, denoted as $Y_{i}=\sum_{c=1}^{p}\sum_{d=1}^{q}P_{i}\left(c,d\right)O\left(c,d\right)$. The modulated patterns are of the same pixel-size p×q with the object image. As specified by the Nyquist–Shannon criterion, we need to perform full sampling, i.e., we should make the number of measurements equal to N=p×q. For instance, the Hadamard and Fourier patterns are two classic deterministic orthogonal bases which are widely used for full sampling. To reduce the sampling ratio, one can use the CS theory by exploiting the sparsity of the object image. The modulated patterns used in compressive sampling scheme are generally 0–1 random, so the overlap between each pattern and the object part will also be random, causing a loss of half flux of photons on average. In addition, the non-negativity of each pattern will generate a DC term in each measured value. Note that one pattern will present as a complementary pair in two reflection directions of the DMD, and we proposed a complementary differential measurement technology in our previous work [26], where two reflection arms are sampled simultaneously by two single-pixel detectors. By this method, the common mode noise can be directly eliminated, the measurement SNR can be increased, and the entire flux of photons can be fully utilized. The two single-pixel detectors can also be replaced by a free-space large-area balanced amplified photodetector (BAP) [37], which can directly output the voltage that is proportional to the difference between the photocurrents in two arms, but without additional synchronization burden. However, we need to set multiple lenses even with multiple plane mirrors in both reflection arms for the sake of symmetrical optical path design. Even with industrialized optomechanical system design, we cannot strictly guarantee that these two light paths are completely the same and the collected spots as well as the collection efficiencies on two photodetectors are the same. Given this, it is hard to ensure that the common mode noise can be eliminated to almost zero. To address this issue, we design a simple secondary complementary balancing method here.

Assume that $Y_{i}^{+}$ and $Y_{i}^{-}$ are two single-pixel values recorded in two reflection directions of the DMD (corresponding to $+12^{\circ }$ and $-12^{\circ }$ orientations of micromirrors, respectively), and can be expressed as

(1) $$Y_{i}^{+}=\sum_{c}\sum_{d}P_{i}^{+}\left(c,d\right)O\left(c,d\right),$$

(2) $$Y_{i}^{-}=\sum_{c}\sum_{d}P_{i}^{-}\left(c,d\right)O\left(c,d\right),$$

where the *i*th complementary random pattern pair ($P_{i}^{+}$ and $P_{i}^{-}$) satisfies $P_{i}^{+}+P_{i}^{-}=𝟙$. Here, 𝟙 denotes a matrix of the same size of $P_{i}^{+}$ and $P_{i}^{-}$ consisting of all ones.

If the object image *O* is flattened into a column vector *x* of N×1, then each modulated pattern can be reshaped into a row vector of 1×N, and *M* such patterns will form a measurement matrix *A* of M×N. The single-pixel values will constitute a sequence of M×1, which can be written as y=Ax+e, where *e* of the same size M×1 stands for the stochastic noise. Generally, we can find the sparse representations of natural object images in some invertible (e.g., orthogonal or approximately orthogonal) or redundant bases Ψ, i.e., $x=\Psi x^{\prime }$. Then, we will have $y=A\Psi x^{\prime }+e$. In the complementary measurement scheme [26], the single-pixel value sequences measured by two photodetectors can be written as

(3) $$y^{+}=A^{+}\Psi x^{\prime }+e^{+},$$

(4) $$y^{-}=A^{-}\Psi x^{\prime }+e^{-},$$

where $A^{+}$ and $A^{-}$ are complementary, all consisting of 0 and 1, $e^{+}$ and $e^{-}$ denote the noise in two reflection arms. Their differential signal can be written as

(5) $$y^{+}-y^{-}=\hat{A}\Psi x^{\prime }+e^{+}-e^{-},$$

where $\hat{A}=A^{+}-A^{-}$. The entries of the differential measurement matrix $\hat{A}$ are ±1 instead of 0 and 1. In the ideal model, we expect the two-arm optical paths to be completely symmetrical, with no luminous flux loss, no difference in propagation distance and collection efficiency, and the environmental noise in two reflection arms is of the same magnitude (i.e., independent and identically distributed). However, in actual measurement, it is difficult for us to acquire the above expected conditions.

To solve this problem, we assume that the single-pixel values measured in the two arms have a linear transformation with the expected values, then we will have

(6) $${y^{\prime }}^{+}=k^{+}\left(A^{+}\Psi x^{\prime }+e^{+}\right)+b^{+},$$

(7) $${y^{\prime }}^{-}=k^{-}\left(A^{-}\Psi x^{\prime }+e^{-}\right)+b^{-},$$

where $k^{+}$ and $k^{-}$ stand for the proportionality coefficients, $b^{+}$ and $b^{-}$ are constant bias. If we directly replace the above modulated patterns with their respective inverse (complementary) patterns, then two single-pixel value sequences received by two photodiodes can be rewritten as:

(8) $${y^{\prime \prime }}^{+}=k^{+}\left(A^{-}\Psi x^{\prime }+e^{+}\right)+b^{+},$$

(9) $${y^{\prime \prime }}^{-}=k^{-}\left(A^{+}\Psi x^{\prime }+e^{-}\right)+b^{-},$$

where the proportionality coefficients and constant bias remain unchanged, but the modulated patterns seen in two detection directions are changed to their inverse ones. In terms of matrix representation, it means that only $A^{+}$ and $A^{-}$ exchange their positions in actual observations.

Since the BAP can automatically output the differential signal of the two arms, the full dynamic range of the BAP can be used to record the positive-negative fluctuations. Then, we will obtain two differential signals for a set of complementary modulation pattern pairs:

(10) $$B_{1}={y^{\prime }}^{+}-{y^{\prime }}^{-}=\left(k^{+}A^{+}-k^{-}A^{-}\right)\Psi x^{\prime }+k^{+}e^{+}-k^{-}e^{-}+b^{+}-b^{-},$$

(11) $$B_{2}={y^{\prime \prime }}^{+}-{y^{\prime \prime }}^{-}=\left(k^{+}A^{-}-k^{-}A^{+}\right)\Psi x^{\prime }+k^{+}e^{+}-k^{-}e^{-}+b^{+}-b^{-}.$$

Here, $B_{1}$ and $B_{2}$ stand for the output signals of the BAP that correspond to complementary modulation pattern pairs. It can be seen that each output of the BAP is actually the differential value of two inputs recorded by its two photodiodes. From these formulas, we can also see that the impact of optical imbalance (i.e., the coefficients $k^{+}$ and $k^{-}$) cannot be negligible, which will incur more complicated noise and degrade the quality of reconstructed images. However, it is interesting to find that the noise items of $B_{1}$ and $B_{2}$ are the same and there are some items in $B_{1}$ and $B_{2}$ that can be mutually merged. Thus, to rebuild the correspondence between the measured values and measurement matrix, we make the difference between these two signals and obtain the following equation:

(12) $$B_{1}-B_{2}=[\left(k^{+}\left(A^{+}-A^{-}\right)-k^{-}\left(A^{-}-A^{+}\right)\right]\Psi x^{\prime }=\hat{A}\left[\left(k^{+}+k^{-}\right)\Psi x^{\prime }\right]=k^{\prime }\hat{A}\Psi x^{\prime },$$

where $k^{\prime }=k^{+}+k^{-}$. It can be seen from this equation that the environmental noise (including the noise $e^{+}$, $e^{-}$ in both reflection arms and the constant biases $b^{+}$, $b^{-}$) is eliminated to the greatest extent, leading to the increase of the measurement SNR and accuracy, and the values of the reconstructed image will be $k^{\prime }$ times those of the original one, leading to an increase in image pixel values but not affecting the actual reconstructed image content. More importantly, by this means, no matter how much the optical difference between two reflection arms is, as long as its impact on the recorded signals is linear, the influence of the optical imbalance can be minimized. In addition, it is worth noting that the differential operations of the first layer are achieved on the BAP device, while the differential operation of the second layer is completed by data post-processing. Given this, we call this method the secondary complementary balancing strategy.

## 3. Simulation

**Figure 1:**
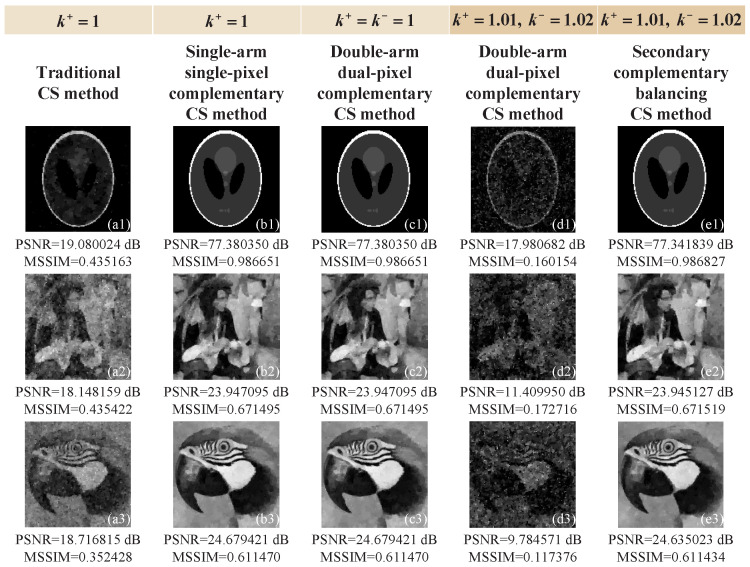
Simulation results of the traditional CS method without complementary measurements (**a1**–**a3**), the single-arm single-pixel complementary CS method (**b1**–**b3**), the double-arm dual-pixel complementary CS method in both an ideal situation (**c1**–**c3**) and imbalanced situation (**d1**–**d3**), and the secondary complementary balancing CS method in the imbalanced case (**e1**–**e3**), respectively. The pixel-sizes of reconstructed images are 128×128 and the total sampling ratios are all 25%.

We first made a comparison between the traditional CS method without complementary measurements, the single-arm single-pixel complementary CS method, the double-arm dual-pixel complementary CS method (in both ideal and imbalanced situations), and the secondary complementary balancing CS method (only in imbalanced situation), and presented their simulation results in Figure 1. All four of these methods applied a total variation minimization (TVAL3) solver [17] to solve the ill-posed underdetermined linear problem (where the number of equations or measurements is less than the number of unknowns). We chose three grayscale standard test images (i.e., head phantom, man, and parrot) of 128×128 pixels as our original images. To make a fair comparison, the total sampling ratios applied for these four methods were all fixed to 25%. To acquire a quantitative measure of the reconstruction performance, the peak signal-to-noise ratio (PSNR) and mean structural similarity (MSSIM) [39] were used here as a figure of merit. The PSNR can evaluate the pixel error between an original image $U_{o}$ and a reconstructed image $\tilde{U}$, defined as $\mathrm{PSNR}=10\log(255^{2}/\mathrm{MSE})$, where $\mathrm{MSE}=\frac{1}{pq}\sum_{c,d=1}^{p,q}{\left[U_{o}\left(c,d\right)-\tilde{U}\left(c,d\right)\right]}^{2}$. However, it does not consider the visual recognition perception characteristics of the human eye. Instead, the MSSIM is a full reference metric, which is based on the assumption that the human eye will extract the structural information when viewing the image. Naturally, the larger the PSNR and MSSIM values are, the better is the image quality.

As we can see from Figure 1a1–b3, the single-arm single-pixel complementary CS method can definitely obtain a better performance than the traditional CS approach when using the same total sampling ratio, and both only use a single-arm light path for detection, thus there is no optical imbalance problem. Utilizing the natural complementary property of the patterns seen in two reflection directions of the DMD, we can convert two successive complementary measurements (in single-arm) into two simultaneous measurements (in double-arm) and acquire the equivalent imaging results under ideal conditions (see Figure 1c1–c3). Thus, both a single-arm single-pixel complementary CS method and double-arm dual-pixel complementary CS method have the same total number of measurements. However, in actual measurement, it is difficult for us to ensure that the optical paths of these two arms are absolutely symmetrical with balanced light intensities. Here, we give an imbalanced case of $k^{+}=1.01$, $k^{-}=1.02$, $b^{+}=1$, $b^{-}=5$ as an example, and the double-arm dual-pixel complementary CS method will present a sharp decline in its reconstruction performance (see Figure 1d1–d3). As can be seen from this example, the image degradation problem caused by the asymmetry in two arms cannot be ignored. It is worth mentioning that $b^{+}$ and $b^{-}$ are constants that will not have much influence on image reconstruction according to the theory of CS, and can be eliminated by our secondary complementary balancing. From Figure 1e1–e3, we can clearly see that, in an imbalanced situation, our secondary complementary balancing CS method can acquire similar imaging performance with both a single-arm single-pixel complementary CS method and a doublearm dual-pixel complementary CS method (in an ideal situation), and much better than that of a double-arm dual-pixel complementary CS method in an imbalanced situation (see Figure 1d1–d3). Therefore, our secondary complementary balancing CS method can effectively eliminate the influence of optical imbalance. In addition, since the differential measurement function of the BAP is used, a set of complementary differential measurements is combined into one balanced measurement. Thus, the complementary modulation will not increase its total number of measurements compared with a conventional complementary CS method.

**Figure 2:**
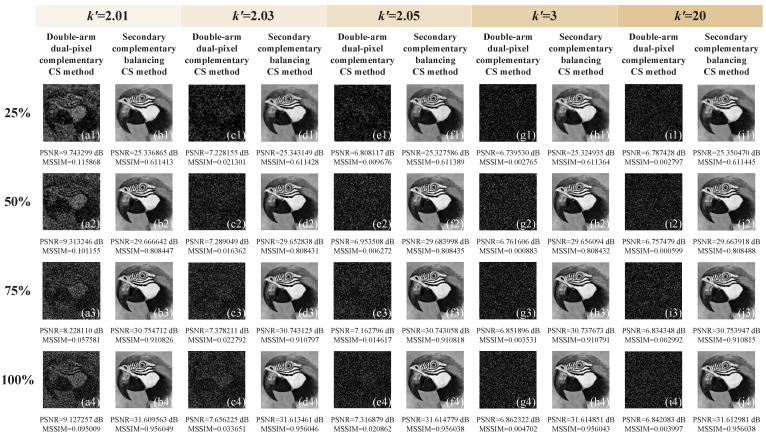
Simulation results of the image parrot by using traditional double-arm dual-pixel complementary CS ((**a1**–**a4**), (**c1**–**c4**), (**e1**–**e4**), (**g1**–**g4**) and (**i1**–**i4**)) and secondary complementary balancing CS method ((**b1**–**b4**), (**d1**–**d4**), (**f1**–**f4**), (**h1**–**h4**) and (**j1**–**j4**)), with different sampling rates 25%, 50%, 75%, and 100% (from **top** to **bottom**) and different imbalance coefficients $k^{\prime }=2.01$, 2.03, 2.05, 3, and 20 (from **left** to **right**).

**Figure 3:**
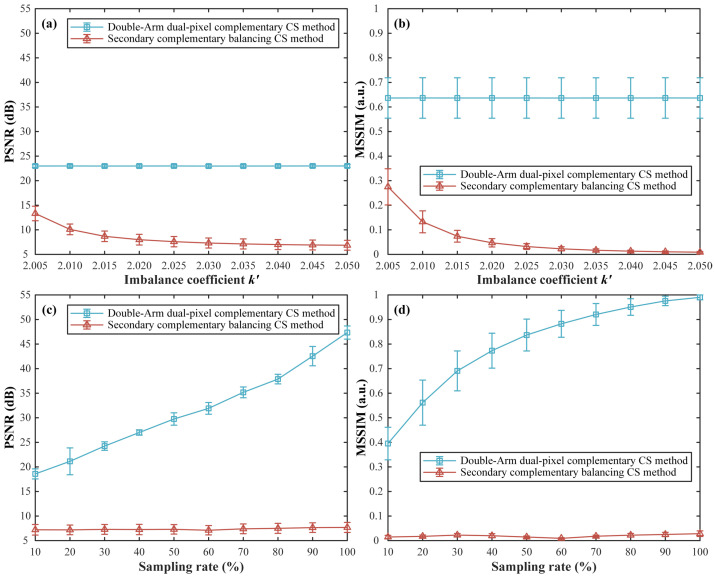
Comparisons of reconstructed results of several general scenes by using the double-arm dual-pixel complementary CS method and the secondary complementary balancing CS method. (**a**,**b**) are the PSNR and MSSIM curves as a function of the imbalance coefficient $k^{\prime }$, respectively. (**c**,**d**) are the PSNR and MSSIM curves as a function of the sampling rate, respectively. In these curves, each point is acquired by averaging PSNR or MSSIM results of five different complex grayscale object scenes, including Barbara, goldhill, sailboat, cablecar and pens, all of 128×128 pixels. The half height of each error bar indicates the standard deviation of each point.

Then, we changed the total sampling ratio and imbalance coefficient $k^{\prime }$ to test the imaging performance. Without loss of generality, we directly set $k^{+}=1$ and assumed that the two reflection arms of the DMD have a difference of 1%, 3%, 5%, 200%, and 1900% in recorded light intensities, respectively, i.e., $k^{\prime }=2.01$, 2.03, 2.05, 3, and 20. As the asymmetry increases, more and more cluttered points appear in the image reconstructed by the traditional double-arm dual-pixel complementary CS method, as shown in Figure 2a1–a4,c1–c4,e1–e4,g1–g4,i1–i4. As we can see from Figure 2b1–b4,d1–d4,f1–f4,h1–h4,j1–j4, our secondary complementary balancing CS method can well suppress the effects caused by the imbalance of the optical paths and acquire a better image quality even at low sampling ratios. For a more intuitive comparison, we further drew the variation curves of the PSNRs and MSSIMs of these two methods with the increase of the imbalance coefficients as well as the increase of the sampling rates, as shown in Figure 3. These curves also demonstrate that our secondary complementary balancing CS method performs better than the conventional double-arm dual-pixel complementary CS method.

**Figure 4:**
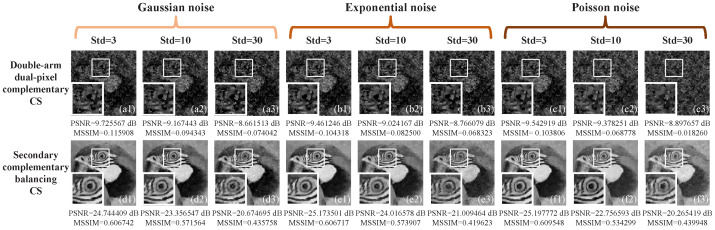
Reconstructed results of 128×128 pixels with 25% sampling rate under additive noise of three kinds of distributions. (**a1**–**f3**) are the results of the double-arm dual-pixel complementary CS method and secondary complementary balancing CS method under the Gaussian, exponentially and Poisson distributed noise, respectively. Here, Std denotes the standard deviation of the noise distribution.

Next, we further investigated the effect of three kinds of additive noise on image quality. Here, we chose the Gaussian, exponentially and Poisson distributed noise, and directly added them onto the measured values. The results were given in Figure 4, where Std stands for the standard deviation of the distribution which the additive noise follows. All images were recovered with 25% sampling rate under the situation of $k^{+}=1$ and $k^{-}=1.01$. From Figure 4a1–a3,b1–b3,c1–c3, we could see that the optical imbalance has a significant degrading effect on the reconstructed images when using double-arm dual-pixel complementary CS method. However, in Figure 4d1–d3,e1–e3,f1–f3, it could be seen that the secondary complementary balancing CS method has a good robustness against measurement noise, with the image details (see the enlarged images marked in the white square frames) being well preserved.

## 4. Experiments

**Figure 5:**
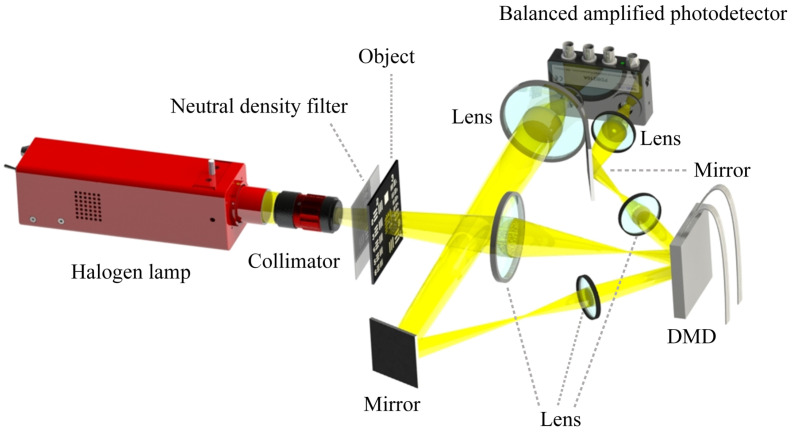
Experimental setup of secondary complementary balancing compressive imaging. DMD: digital micromirror device.

Our experimental setup is given in Figure 5: the thermal light (ranging from 360 nm to 2600 nm) emitted from a halogen lamp is collimated by a collimator and attenuated by some 2 inch × 2 inch neutral density filters to the ultra-weak light level, and then illuminates an object (a negative 1951 USAF resolution test chart of 3 inch × 3 inch). The transmitted light is projected vertically via an imaging lens onto a 0.7 inch DMD consisting of 768×1024 micromirrors (i.e., pixels), which is encoded with pre-prepared complementary patterns. The reflected light of the DMD is emitted at angles of $\pm 24^{\circ }$ with respect to the normal of the DMD’s work plane. The light from two reflection arms separately passes through two sets of lenses and a mirror, and is collected by a BAP (Thorlabs PDB210A/M), which will directly output the differential signal of two photodiodes’ photocurrents. The output voltage signal will be fully recorded by a mixed signal oscilloscope (Tektronix MSO64 6-BW-4000). In our experimental setup, the light source is always on, the BAP has a sampling frequency up to 5 MHz, and the oscilloscope used owns a high sampling frequency of 24 GS/s, both are much higher than the top working frequency of the DMD (22 KHz). Therefore, the upper limit of the sampling speed of the whole experimental setup is mainly determined by the DMD. When we apply 5% sampling ratio and 100% sampling ratio for an original image of 48×48 pixels, it only takes us 0.005 s and 0.1 s to sample the data, and generally lower than 0.3 s to reconstruct the images with a regular laptop. Since the BAP used here cannot work in single-photon level, a too high sampling ratio will drown the signal in noise. Thus, without loss of generality, in the experiment, we set the DMD’s switching frequency to 300 Hz; then, the sampling time with 5% sampling ratio is 0.384 s, which is still within the acceptable range. It is worth mentioning that this switching frequency can be set higher for real-time application.

**Figure 6:**
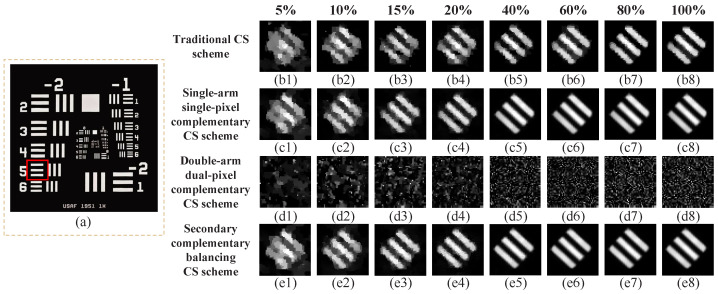
Experimental results of four CS schemes using different sampling rates (5%, 10%, 15%, 20%, 40%, 60%, 80% and 100% from left to right) in the imbalanced case. (**a**) The stripe pattern marked in the red square of the negative 1951 USAF resolution test chart is treated as the original object. (**b1**–**e8**) are the results of the traditional CS scheme without complementary measurements, the singlearm single-pixel complementary measurement scheme, the double-arm dual-pixel complementary measurement scheme, and the secondary complementary balancing CS scheme, respectively.

In the experiment, we also tested the practical performance of a traditional CS scheme without complementary measurements, a single-arm single-pixel complementary CS scheme, a double-arm dual-pixel complementary CS scheme, and a secondary complementary balancing CS scheme. Here, the complementary patterns were generated from the random patterns used in the first method. Since the BAP has two additional monitor ports (denoted as MONITOR+ and MONITOR-), which can independently output the photocurrents of two photodetectors, a traditional CS scheme without complementary measurements can be performed by using the light intensities (in one reflection direction) collected by one photodetector of the BAP. Since the patterns seen in two reflection directions of the DMD were exactly complementary, we made a difference between the photocurrents detected by two photodetectors. By this means, a traditional double-arm dual-pixel complementary measurement scheme [26,27] was realized. Then, we made the DMD modulate one random pattern immediately followed by its inverse/complementary one, and used the photocurrents provided by one monitor output port of the BAP, which corresponds to one reflection arm, to realize single-arm single-pixel complementary measurement [29,30,32]. While in our secondary complementary balancing CS scheme, the voltage proportional to the difference between the photocurrents in two arms was used directly for image reconstruction, and the complementary modulation was also applied. The patterns encoded onto the DMD were of 48×48 pixel-units, each of which would occupy 12×12=144 micromirrors of the DMD, i.e., a total of 576×576 micromirrors were actually involved in the optical modulation. The experimental results of these four schemes under different sampling ratios (ranging from 5% to 100%) were presented in Figure 6. As shown in Figure 6a, the red square marked in the negative 1951 USAF resolution test chart was chosen here as the original image, whose stripes are separated by 1.26 mm on the resolution test chart. It can be clearly seen that the imaging quality of a traditional CS scheme, a single-arm single-pixel complementary measurement scheme, and a secondary complementary balancing CS scheme improves with the increase of the sampling ratio. Due to the optical asymmetry, the double-arm dual-pixel complementary measurement scheme could not acquire the image quality improvement as the sampling ratio increases. The reconstruction qualities of our secondary complementary balancing CS scheme (see Figure 6(e1–e8)) are much better than those of the traditional CS scheme (see Figure 6(b1–b8)) and the traditional double-arm dual-pixel complementary measurement scheme (see Figure 6(d1–d8)), and are similar to those of the single-arm single-pixel complementary measurement scheme (see Figure 6(c1–c8)), at any sampling ratios. However, actually, there was no optical imbalance in the single-arm single-pixel complementary measurement scheme, and taking its experimental results as a reference mainly considered that it also used complementary modulation. In addition, when the sampling rate reached 40%, the image quality of this proposed method was almost the same as that of the traditional CS scheme without complementary measurements under full sampling conditions. These results are consistent with the simulation results. Therefore, our secondary complementary balancing CS scheme can effectively eliminate the image degradation problem caused by optical imbalance in dual-arm measurements.

**Figure 7:**
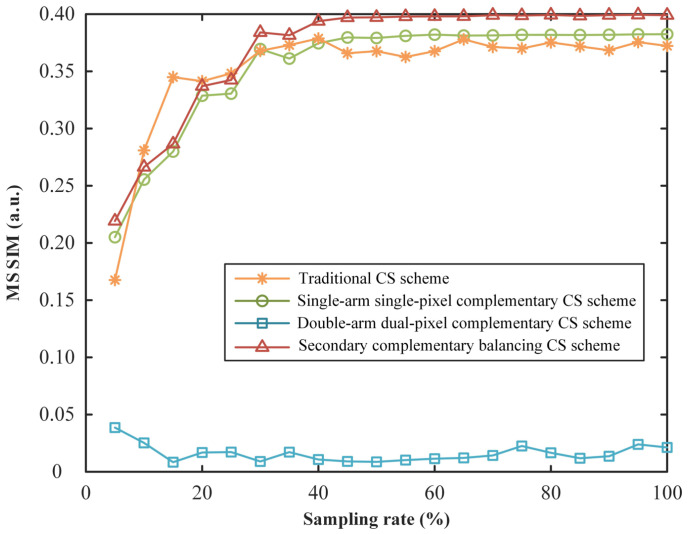
Curves of MSSIM values of a traditional CS scheme, single-arm single-pixel complementary CS scheme, double-arm dual-pixel complementary CS scheme and secondary complementary balancing CS scheme as a function of the sampling rate.

Next, we further analyzed the performance of the above four kinds of schemes, by plotting the MSSIM curves as a function of the sampling ratio as shown in Figure 7. From the curves, we can see that, when the sampling rate is higher than 40%, our method outperforms the others. Although there is only one collecting light path (without optical imbalance problem) in both the traditional CS scheme and single-arm single-pixel complementary CS scheme, their reconstruction qualities are still slightly lower than those of our secondary complementary balancing CS scheme, especially at high sampling rates. This is because our secondary complementary balancing strategy can not only eliminate optical imbalance but also suppress measurement noise by noise balancing. It should be noted here that, in the curves as shown in Figure 7, the MSSIM values of the traditional CS scheme present some fluctuation at low sampling rates, which is actually an estimation deviation caused by the fact that the MSSIM as a full-parameter image quality metric relies too much on pixel values for image evaluation. From Figure 6, we can clearly observe that the visibility of the reconstructed images via a double-arm dual-pixel complementary CS scheme is always lower than those of the other three schemes at any sampling rate under optical imbalance conditions. By calculation, the average MSSIM of our proposed scheme is 23.1716 times that of a double-arm dual-pixel complementary CS scheme.

**Figure 8:**
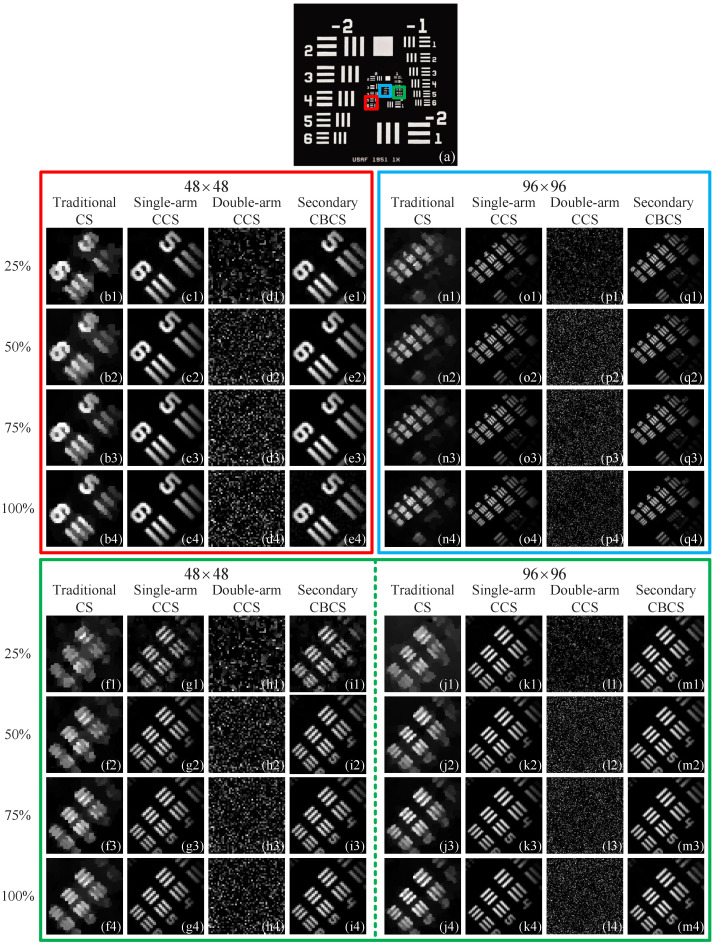
Reconstructed results of four CS schemes for different regions of the resolution test chart with different sampling rates. (**a**) gives three imaging regions in the resolution test chart successively marked with red, green and blue boxes. (**b1**–**e4**) are the recovered images of the region inside the red box of (**a**) with modulated patterns of 48×48 pixel-units by using four CS schemes, respectively. (**f1**–**m4**) are the restored images of the region within the green box of (**a**) with modulated patterns of 48×48 and 96×96 pixel-units, respectively. (**n1**–**q4**) are the retrieved images of the region in the blue box of (**a**) with modulated patterns of 96×96 pixel-units also by using four CS schemes, respectively. The abbreviations traditional CS, single-arm CCS, double-arm CCS, and secondary CBCS in the figure stand for a traditional CS scheme without complementary measurements, a singlearm single-pixel complementary measurement scheme, a double-arm dual-pixel complementary measurement scheme, and a secondary complementary balancing CS scheme, respectively.

Then, in Figure 8, we further compared the imaging performance between our secondary complementary balancing CS scheme and other three CS schemes in different imaging regions of the resolution test chart and in different spatial resolutions. Figure 8b1–b4,c1–c4,d1–d4,e1–e4 are the results of the original image region marked in the red square of Figure 8a by using four CS schemes and different sampling ratios (changing from 25% to 100%), with the modulated patterns of 48×48-pixel-units. Note that the red square is located in Group 0, and the parallel lines for Elements 5 and 6 are 1574.90 µm and 1403.08 µm long, 314.98 µm and 280.62 µm wide, separated by equal spaces of 314.98 µm and 280.62 µm wide, respectively. It can be seen that the results of the single-arm singlepixel complementary CS scheme (see Figure 8c1–c4) and our secondary complementary balancing CS scheme (see Figure 8e1–e4) are all much better than those of the traditional CS scheme without complementary measurements (see Figure 8b1–b4) and double-arm dual-pixel complementary measurement scheme (see Figure 8d1–d4), with the same sampling ratios. In this experiment, the sampling rate of 25% is enough to clearly reconstruct the images. From Figure 8d1–d4,e1–e4, we can see that, when there exists optical imbalance in double reflection arms of the DMD, the double-arm dual-pixel complementary measurement scheme does not work at all, while our secondary complementary balancing CS scheme can still acquire high quality restored images and is not affected by optical imbalance. Then, we changed the imaging region to the green square of Figure 8a and used modulated patterns of 48×48 and 96×96 pixel-units. The corresponding results are given in Figure 8f1–i4,j1–m4. Since the green square is in Group 1, the parallel lines for Elements 4, 5, and 6 are 883.88 µm, 787.45 µm and 701.54 µm long, 176.78 µm, 157.49 µm and 140.31 µm wide, separated by equal spaces of 176.78 µm, 157.49 µm and 140.31 µm wide, respectively. For elements in this group, the modulated patterns of 48×48 pixel-units caused slightly blurred reconstructions at a 25% sampling rate, but the reconstructed images could be much clearer with a 50% sampling rate. When we used the modulated patterns of 96×96 pixel-units, using a 25% sampling rate can achieve excellent reconstructed image quality. It can be seen that, as the pixel resolution of the modulated patterns increases, the quality of recovered images will be better. Thus, the pixel resolution of the modulated patterns is an important parameter that affects the image quality, but its increase will also incur an increase in computation time and memory consumption, hence a trade-off needs to be made. Next, we further shifted the imaging region to the blue square of Figure 8a, Elements 2–6 in Group 2 have line widths of 111.36 µm, 99.21 µm, 88.39 µm, 78.75 µm, and 70.15 µm, respectively. In Figure 8(n1–n4), the spacing of these parallel lines is difficult to distinguish by using a traditional CS scheme without complementary measurements. In contrast, our secondary complementary balancing CS scheme (see Figure 8q1–q4) can acquire almost the same image quality with a single-arm single-pixel complementary measurement scheme (see Figure 8(o1–o4)), with the parallel lines being fully distinguishable. However, in a double-arm dual-pixel complementary measurement scheme, it still cannot obtain any useful object information.

It is worth mentioning that the use of random patterns in this paper is just for demonstrations, and in fact the modulated patterns are not limited to this. Since the modulated patterns in balanced detection scheme need to be differentiated, the measurement matrix is positive-negative distributed. Thus, the Hadamard basis patterns that consist of ±1 will be another option. For example, the popular optimization sorting (including Russian dolls sorting [21], cake-cutting sorting [22], and origami sorting [23]) of the Hadamard basis in recent years can be also used in this secondary complementary balancing scheme to further reduce the sampling ratios required to obtain high image quality. Since this is not the focus of this paper, it will not be described in detail in this paper.

## 5. Conclusions

In this paper, a secondary complementary balancing mechanism is proposed and combined with CS to eliminate the effect of optical asymmetry (imbalance) on image reconstruction degradation that exists in double-arm balanced (differential) detection of the complementary SPI scheme. Here, we use a free-space BAP to perform balanced measurements. By making the DMD modulate a pattern immediately followed by its complementary one, we can achieve a good cancellation of DC components of detected values as well as a high-quality image reconstruction. The use of secondary complementary balancing compressive measurement can improve the measurement SNR and accuracy by amplifying the fluctuating portions of the measured values and making full use of an entire dynamic range of BAP to record these positive-negative fluctuations. The simulation results have demonstrated that, even if there is only a small amount of optical imbalance (with a small imbalance coefficient $k^{\prime }$) in two reflection light paths of the DMD, it will have a great degrading effect on the imaging results of the traditional double-arm dual-pixel complementary measurement scheme. By using our secondary complementary balancing mechanism, the impact of optical imbalance can be eliminated, even in the presence of severe noise of different distributions. The experiment results further verified the feasibility of the proposed method, and proved that our secondary complementary balancing CS scheme performs slightly better than the single-arm single-pixel complementary measurement scheme (which is free from the trouble of optical imbalance) and the average MSSIM value of our scheme is over 20 times that of the double-arm dual-pixel complementary CS scheme under optical imbalance conditions. In the future, this secondary complementary balancing method can be combined with the iterative denoising method and deterministic modulated patterns (such as optimally ordered Hadamard matrix, Fourier matrix), and we expect to apply it to moving target tracking, full-color imaging, multispectral imaging, biological microscopy, and secure communication. Furthermore, it can also provide inspiration for any balanced detection application scenarios where optical imbalance exists. We believe that this mechanism will be very helpful in double-arm complementary SPI and may offer many benefits, especially for balanced detection.

## References

[1] Duarte M.F., Davenport M.A., Takhar D., Laska J.N., Sun T., Kelly K.F., Baraniuk R.G. (2008). Single-pixel imaging via compressive sampling. IEEE Sig. Proc. Mag..

[2] Edgar M.P., Gibson G.M., Padgett M.J. (2019). Principles and prospects for single-pixel imaging. Nat. Photonics.

[3] Gibson G.M., Johnson S.D., Padgett M.J. (2020). Single-pixel imaging 12 years on: A review. Opt. Express.

[4] Shapiro J.H. (2008). Computational ghost imaging. Phys. Rev. A.

[5] Katz O., Bromberg Y., Silberberg Y. (2009). Compressive ghost imaging. Appl. Phys. Lett..

[6] Yang J.-Z., Li M.-F., Chen X.-X., Yu W.-K., Zhang A.-N. (2020). Single-photon quantum imaging via single-photon illumination. Appl. Phys. Lett..

[7] Durán V., Clemente P., Fernádez-Alonso M., Tajahuerce E., Lancis J. (2012). Single-pixel polarimetric imaging. Opt. Lett..

[8] Deng Q., Zhang Z., Zhong J. (2020). Image-free real time 3D tracking of a fast-moving object using dual-pixel detection. Opt. Lett..

[9] Pian Q., Yao R., Sinsuebphon N., Intes X. (2017). Compressive hyperspectral time-resolved wide-field fluorescence lifetime imaging. Nat. Photon..

[10] Studer V., Bobin J., Chahid M., Moussavi H.S., Candès E.J., Dahan M. (2012). Compressive fluorescence microscopy for biological and hyperspectral imaging. Proc. Natl. Acad. Sci. USA.

[11] Graff C.G., Sidky E.Y. (2015). Compressive sensing in medical imaging. Appl. Opt..

[12] Wu D., Luo J., Huang G., Feng Y., Feng X., Zhang R., Shen Y., Li Z. (2021). Imaging biological tissue with high-throughput single-pixel compressive holography. Nat. Commun..

[13] Durán V., Soldvilla F., Irles E., Clemente P., Tajahuerce E., Andrés P., Lancis J. (2015). Compressive imaging in scattering media. Opt. Express.

[14] Yu W.-K. (2019). Cryptographic key distribution over a public network via variance-based watermarking in compressive measurements. Appl. Opt..

[15] Donoho D.L. (1992). Superresolution via sparsity constraints. SIAM J. Math. Anal..

[16] Donoho D.L. (2006). Compressed sensing. IEEE Trans. Inf. Theory.

[17] Li C.B. (2010). An Efficient Algorithm for Total Variation Regularization with Applications to the Single Pixel Camera and Compressive Sensing. Master’s Thesis.

[18] Gatti A., Brambilla E., Bache M., Lugiato L.A. (2004). Ghost imaging with thermal light: Comparing entanglement and classical correlation. Phys. Rev. Lett..

[19] Ferri F., Magatti D., Lugiato L.A., Gatti A. (2010). Differential ghost imaging. Phys. Rev. Lett..

[20] Xu Z.-H., Chen W., Penuelas J., Padgett M., Sun M.-J. (2018). 1000 fps computational ghost imaging using led-based structured illumination. Opt. Express.

[21] Sun M.-J., Meng L.-T., Edgar M.P., Padgett M.J., Radwell N. (2017). A Russian dolls ordering of the Hadamard basis for compressive single-pixel imaging. Sci. Rep..

[22] Yu W.-K. (2019). Super sub-Nyquist single-pixel imaging by means of cake-cutting Hadamard basis sort. Sensors.

[23] Yu W.-K., Liu Y.-M. (2019). Single-pixel imaging with origami pattern construction. Sensors.

[24] Vaz P.G., Amaral D., Ferreira L.F.R., Morgado M., Cardoso J. (2020). Image quality of compressive single-pixel imaging using different Hadamard orderings. Opt. Express.

[25] Zhang Z., Ma X., Zhong J. (2015). Single-pixel imaging by means of Fourier spectrum acquisition. Nat. Commun..

[26] Yu W.-K., Liu X.-F., Yao X.-R., Wang C., Zhai Y., Zhai G.-J. (2014). Complementary compressive imaging for the telescopic system. Sci. Rep..

[27] Radwell N., Mitchell K.J., Gibson G.M., Edgar M.P., Bowman R., Padgett M.J. (2014). Single-pixel infrared and visible microscope. Optica.

[28] Zhou D., Cao J., Cui H., Hao Q., Chen B.-K., Lin K. (2021). Complementary Fourier single-pixel imaging. Sensors.

[29] Yu W.-K., Yao X.-R., Liu X.-F., Li L.-Z., Zhai G.-J. (2015). Three-dimensional single-pixel compressive reflectivity imaging based on complementary modulation. Appl. Opt..

[30] Yu W.-K., Yao X.-R., Liu X.-F., Li L.-Z., Zhai G.-J. (2015). Compressive moving target tracking with thermal light based on complementary sampling. Appl. Opt..

[31] Welsh S.S., Edgar M.P., Bowman R., Sun B.Q., Padgett M.J. (2015). Near video-rate linear Stokes imaging with single-pixel detectors. J. Opt..

[32] Yu W.-K., Yao X.-R., Liu X.-F., Lan R.-M., Wu L.-A., Zhai G.-J., Zhao Q. (2016). Compressive microscopic imaging with ”positivenegative” light modulation. Opt. Commun..

[33] Luo B.-B., Tsai K.-C., Liu J.-P. Computational ghost imaging by using complementary illumination patterns. Proceedings of the SPIE 10711, Biomedical Imaging and Sensing Conference.

[34] Welsh S.S., Edgar M.P., Bowman R., Jonathan P., Sun B., Padgett M.J. (2013). Fast full-color computational imaging with single-pixel detectors. Opt. Express.

[35] Edgar M.P., Gibson G.M., Bowman R.W., Sun B., Radwell N., Mitchell K.J., Welsh S.S., Padgett M.J. (2015). Simultaneous real-time visible and infrared video with single-pixel detectors. Sci. Rep..

[36] Soldevila F., Clementte P., Tajahuerce E., Uribe-Patarroyo N., Andrés P., Lancis J. (2016). Computational imaging with a balanced detector. Sci. Rep..

[37] Denk O., Musiienko A., Žídek K. (2019). Differential single-pixel camera enabling low-cost microscopy in near-infrared spectral region. Opt. Express.

[38] Carleton H.R., Maloney W.T. (1968). A balanced optical heterodyne detector. Appl. Opt..

[39] Wang Z., Bovik A.C., Sheikh H.R., Simoncelli E.P. (2004). Image quality assessment: From error visibility to structural similarity. IEEE Trans. Image Process..

